# Escalating Bi-Directional Feedback Loops between Proinflammatory Microglia and Mitochondria in Ageing and Post-Diagnosis of Parkinson’s Disease

**DOI:** 10.3390/antiox12051117

**Published:** 2023-05-18

**Authors:** Shane Michael Ravenhill, Andrew Howard Evans, Sheila Gillard Crewther

**Affiliations:** 1 School of Psychology and Public Health, La Trobe University, Bundoora 3083, Australia; 2 Department of Medicine, The Walter and Eliza Hall Institute of Medical Research, Melbourne 3052, Australia; 3 Epworth Hospital, Richmond 3121, Australia; 4 Department of Neurology, Royal Melbourne Hospital, Melbourne 3050, Australia

**Keywords:** alpha-synuclein, adenosine triphosphate, bioenergetic capacity, cytokines, dopamine neurons, homeostasis, microglia, mitochondrial quality control, neurodegenerative progression, neuroinflammation, oxidative respiration, oxidative stress, Parkinson’s disease, phagocytosis, proinflammatory immune response, reactive oxygen species

## Abstract

Parkinson’s disease (PD) is a chronic and progressive age-related neurodegenerative disease affecting up to 3% of the global population over 65 years of age. Currently, the underlying physiological aetiology of PD is unknown. However, the diagnosed disorder shares many common non-motor symptoms associated with ageing-related neurodegenerative disease progression, such as neuroinflammation, microglial activation, neuronal mitochondrial impairment, and chronic autonomic nervous system dysfunction. Clinical PD has been linked to many interrelated biological and molecular processes, such as escalating proinflammatory immune responses, mitochondrial impairment, lower adenosine triphosphate (ATP) availability, increasing release of neurotoxic reactive oxygen species (ROS), impaired blood brain barrier integrity, chronic activation of microglia, and damage to dopaminergic neurons consistently associated with motor and cognitive decline. Prodromal PD has also been associated with orthostatic hypotension and many other age-related impairments, such as sleep disruption, impaired gut microbiome, and constipation. Thus, this review aimed to present evidence linking mitochondrial dysfunction, including elevated oxidative stress, ROS, and impaired cellular energy production, with the overactivation and escalation of a microglial-mediated proinflammatory immune response as naturally occurring and damaging interlinked bidirectional and self-perpetuating cycles that share common pathological processes in ageing and PD. We propose that both chronic inflammation, microglial activation, and neuronal mitochondrial impairment should be considered as concurrently influencing each other along a continuum rather than as separate and isolated linear metabolic events that affect specific aspects of neural processing and brain function.

## 1. Introduction

**Figure 1:**
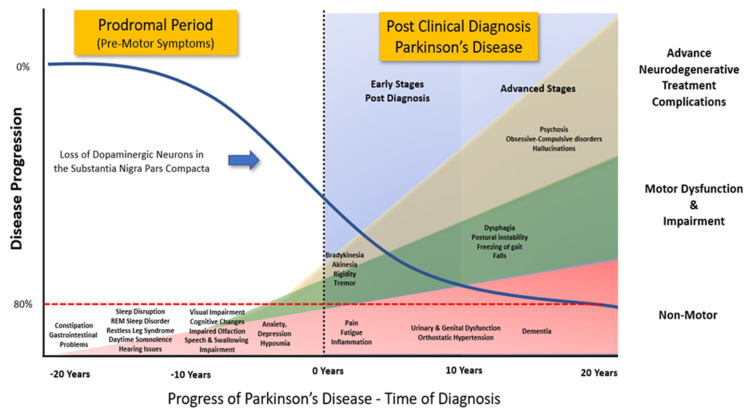
Progression of clinical symptoms from early prodromal through to clinical diagnosis of Parkinson’s disease. The clinical diagnosis of Parkinson’s disease accepts that there is a potential loss of up to 80% of the dopaminergic neurons in the Substantia Nigra Pars Compacta. However, prior to the emergence of any significant motor impairment, a wide variety of symptoms associated with non-motor dysfunction and disability usually precede the clinical diagnosis of Parkinson’s disease by 10–20 years. Adapted from data and figures in Kalia et al. [30], and Tansey et al. [3].

Parkinson’s disease (PD) is an age-related chronic, progressive, multi-system [1,2,3], neurodegenerative disease [4] with an incidence second only to Alzheimer’s disease [1]. A PD diagnosis requires the presence of two core motor features, including diminished movement (bradykinesia), tremor, muscle rigidity, or postural instability. Other behavioural symptoms can include difficulty initiating voluntary movement (akinesia), involuntary eye movements, and blinking [5,6,7,8]. In some cases, it may take up to 15 years before an accurate and reliable clinical diagnosis can be made [9,10,11], even though as many as 50% of PD patients [12] may have been experiencing other pathological changes associated with PD, such as autonomic nervous system dysfunctions [13], REM sleep behaviour disorder [14,15], daytime somnolence, fatigue, depression, and anxiety, orthostatic hypotension [16,17], persistent constipation [18], changes in gut-brain associations [16,19,20] and cognitive decline. Figure 1 illustrates the various clinical symptoms associated with the PD prodromal period through a clinical diagnosis of PD. Data gathered from human post-mortem results indicates that individuals are likely to have lost between 60 and 80% of dopaminergic neurons in the substantia nigra pars compacta (SNpC) at the time of clinical diagnosis [5,8,21]. However, this morphological change is confounding as a potential biomarker, as ageing *per se* is a key risk factor in most neurodegenerative diseases, and cell loss in a normal brain occurs first in mid-life in the Locus Coeruleus, SNpC, and Ventral Tegmentum Area (VTA). These same noradrenergic/dopaminergic areas are also the first in most brains to show increased levels of microglial activation, where microglia are the specialised immune macrophages of the brain [22].

Indeed, Franceschi et al. [23] argue that beyond 40 years of age, immunosenescence results in a gradual and consistent rise in cumulative low-level chronic inflammation [24,25] and the beginning of neuronal loss, henceforth referred to as inflammaging. These changes coincide with a deterioration in the immune system’s capacity to respond efficiently to pathogenic removal and impaired management and control of cellular homeostasis [26]. Age is also linked to increased genomic instability, epigenetic change, mitochondrial dysfunction, impairment, and failure, as well as chronic levels of localised concentrations of proinflammatory microglia [22]. The impact of such impairments and cellular dysfunction triggers ongoing and sustained cytokine storms at neurotoxic levels, resulting in stem cell depletion and impairment of cell signalling and communications [27], and is linked to cognitive decline [28,29].

The aim of the first section of this review is to examine evidence connecting the independent impact of ageing and general inflammatory/inflammaging, (age-related inflammation) responses and the subsequent triggering of a proinflammatory brain-based microglial-mediated immune response in the well-accepted mitochondrial dysfunction in PD. The aim of the second section of the review is to examine molecular evidence documenting the inter-related mechanisms of mitochondrial dysfunction, elevated oxidative stress, increased production of free radicals such as reactive oxygen species (ROS), and impaired adenosine triphosphate (ATP) energy production, resulting in an escalation in microglial-mediated proinflammatory immune responses. Such elevated immune responses are also apparent in many other human diseases, such as cancer, diabetes, multiple sclerosis, cardiovascular disease, and various psychiatric disorders, such as anxiety and depression [31,32,33,34,35,36,37,38].

## 2. Epidemiology of PD

On a global scale, PD is estimated to be the fastest-growing neurological disorder [39,40,41]. The reason for this is multifactorial. Longer lifespans from improved population health measures and better disease treatment lead to longer exposures to environmental toxins [39,42,43,44,45]. Parkinson’s disease affects around 3% of the global population over 65 years of age [41,46] and rises to 5% in people over 85 years [7], i.e., ~12.5 million globally [47,48]. Longitudinal studies also suggest that the socioeconomic burden of PD is increasing globally [49,50], with the Global Burden of Disease Study in 2016 predicting up to a 12% rise in PD rates by 2040 [51,52,53]. Such a rapid growth in PD will not only increase the personal strain on caregivers but also bring adverse health and socio-economic consequences for the economy in general [54]. More recently, the secondary and longer-term effects of SARS-CoV-2 infection have also been predicted to globally increase the incidence rates of long-term neurological and neuropsychiatric complications [55,56,57].

Currently, there is no cure for PD [58], and only palliative medical treatments are available to slow further neurodegenerative damage and disease progression [59,60], highlighting the urgent need to better understand the complex underlying biological processes and identify novel avenues for earlier diagnosis and potential therapeutic intervention to slow progression and severity [61].

## 3. Loss of Dopaminergic and Adrenergic Neurons in PD

The loss of dopaminergic neurons in the SNpC has long been associated with impaired motor movement and cognitive impairment and is usually considered the core pathological characteristic of PD [12,62,63,64]. However, more recent evidence from patients and clinical studies reveals that the pathology of PD follows a caudo-rostral pattern in which the loss of neurons in the locus coeruleus (LC) occurs earlier than the loss of neurons in the SNpC [10,65,66,67]. The clinical effects of monoaminergic cell loss can be predicted preclinically and are supported by recent work on mouse models [68]. The LC is a small brainstem nucleus located in the pons in the CNS and is responsible for producing the neurotransmitter norepinephrine (NE) [69]. When the LC is damaged, individuals show impaired functions similar to PD symptoms, such as sleep disorders, depression, and autonomic nervous dysfunction, long before the emergence of motor impairment in the SNpC [70]. The LC is also involved in managing sleep-wake cycles, memory, learning, alertness, and stress management, while the neurotransmitter NE plays a generalised anti-inflammatory and neuroprotective role. Subsequently, the loss of NE neurons in the LC may trigger microglial activity in the midbrain, increase proinflammatory cytokine release, and potentially contribute to early dopaminergic neuronal loss in the SNpC [71] as a significant aetiological mechanism in the early development of PD. Interestingly, loss of LC NE neurons, like dopaminergic neurons with intra-neuronal cytoplasmic inclusions in the SNpC, also creates Lewy bodies (LB) [72] and Lewy neurites [73]. Indeed, Lewy bodies contain large amounts of aggregated Alpha-Synuclein α-Syn [74,75] which is a presynaptic structural protein that plays a role in the regulation of the synaptic vesicle cycle within the cell [76]. When α-Syn is abnormally aggregated in the neuron, increasing microglial activation as part of the immune response elevates neuroinflammation and activates higher levels of toxic Nicotinamide Adenine Dinucleotide Phosphate (NADPH) oxidase [77] located in the mitochondria. Higher levels of NADPH also leads to increased production of ROS that are neurotoxic and associated with faster rates of SNpC dopaminergic neuronal loss in PD patients compared to healthy controls [78]. As PD advances, dopaminergic neuronal loss occurs most prominently in the SNpC A9 cell group (76% loss) [5,79,80,81], with the neurons located dorsally in SNpC appearing to be less impacted [21]. Results from immunohistochemical analysis in rats and human imaging studies, which allow an accurate quantification of dopaminergic neuronal cell densities, reveal that similar dopaminergic cell losses also occur within the A8 mid-brain retrorubral (31% loss) and A10 ventral tegmentum areas (VTA) (55% loss) [5,21,82], with almost no loss occurring in the central grey matter areas [83]. This differential loss has been explained by observations that dopaminergic neurons in the SNpC are highly vulnerable to impairment due to their higher bioenergetic needs and greater axonal arborizations than comparable dopaminergic neurons in the proximally located VTA. Not unexpectedly, larger axonal arborization and axon terminals require more axonal mitochondria and higher mitochondrial basal oxidative phosphorylation (OXPHOS) rates. The increased mitochondrial requirement also has a concomitant elevation in associated oxidative stress levels, extensive calcium (Ca^2+^) influx requirements, smaller ATP reserve capacity, more complex axonal arborization, and the requirement of a higher concentration of axonal terminal mitochondria to support significantly higher energetic demands compared to dopaminergic neurons in other parts of the brain [84]. Given also that ageing is the major risk factor for PD and has long been related to chronic inflammation, increased mutations in mitochondrial DNA (mtDNA), reduced concentration and number of mitochondria per cell, combined with reduced mitochondrial efficiency in ATP production associated with experimental data supporting the mitochondrial free radical theory of ageing [85], it is necessary to discuss the differences between mitochondria in neurotypicals and those diagnosed with PD [86,87].

## 4. Mitochondrial Function in Ageing and PD

**Figure 2:**
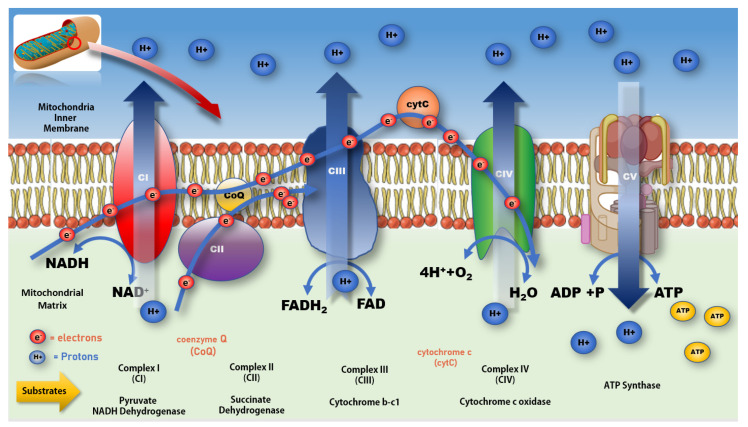
Mitochondrial electron transport chain and production of ATP. Impeding the flow of electrons along the ETC at CI increases the production of free radicals, resulting in a rise in oxidative stress and a lower production of ATP at CV. A more complete review of mitochondrial bioenergetic function and associated impacts on various disease processes can be found in Protasoni et al. 2021 [125].

Most eukaryotic cells, apart from red blood cells, contain mitochondria, where the production of cellular ATP energy occurs via oxidative phosphorylation (OXPHOS) and glycolysis, also known as the citric acid or Krebs cycle. Mitochondria differ from most cell organelles by having a unique genome, an inner and outer membrane, and reproducing by binary fission. Mitochondria not only play a major cellular role in the production of ATP but also store calcium for cell signalling, generate heat, and mediate cell growth and death [88,89,90,91]. The matrix of the mitochondria contains 37 specialised genes of the mitochondrial DNA and the enzymes of the tricarboxylic citric acid (TCA or Krebs’s) cycle [92,93] required for metabolism of the by-products needed for the inner membrane electron transport chain (ETC) processes of OXPHOS via the five multiprotein sub-unit complexes, CI–CV [94,95]. See Figure 2. The ATP energy derived from OXPHOS and glucose metabolism (glycolysis) supports the various metabolic functions that maintain cellular health, homeostasis, and the cells survival within a constantly changing metabolic environment [96]. In addition to energy production, mitochondria play a critical role in managing cellular homeostasis [97], including cell signalling [98], epigenetic regulation [99], Ca^2+^ buffering [100,101,102], activation of proteases and phospholipases [103], heme/iron biosynthesis [104,105], and managing free radical levels [106] by controlling ROS production [107], modulation, and sequestration [108].

Mitochondria also play an important role in providing energy for maintaining protein folding [102], cell division, proliferation, growth, migration [109], innate cellular immune function [110,111,112], and the initiation of apoptotic cell death [107,113]. However, ageing complicates these processes, as both mitochondrial number and function decline with age while mitochondrial DNA mutations increase [114]. Proton leakage associated with the ETC during OXPHOS also escalates with age, raising the levels of free radicals, which become increasingly toxic to the mitochondria [100,115], significantly impacting ATP production and mitochondrial membrane integrity [116] (see Figure 2). In normal, non-aged individuals, the electrons that pass along the ETC produce cellular energy in the form of ATP [111]. During this process, approximately 2% of protons escape from the ETC at complex I CI [117,118] and complex III CIII [109], forming superoxide (O2•−) [119], which is the main building block for free radicals such as ROS.

Mitochondria are dynamic organelles responding to changes in the cell’s physiological and molecular energy environment by continuously balancing between fission and fusion, where fission refers to the ability of the mitochondria to fuse with other mitochondria to form larger or elongated organelles and divide into new mitochondria, and fusion refers to the process whereby the contents of damaged mitochondria are aggregated and recycled into other existing mitochondria [120,121]. Fission is triggered by the dynamin-related protein 1 (Drp1) that is responsible for creating new mitochondria, maintaining their numbers [113], and locating and removing damaged mitochondria via mitophagy [122,123,124]. Fusion is thought to promote more efficient distribution of mtDNA by identifying and removing damaged mitochondria and mixing/diffusing the damaged contents into surrounding healthy mitochondria [119,124].

Functional attributes, distribution, concentration, size, number, and homeostasis of mitochondria are managed by a continuous and complex balance between the age of the individual mitochondria, the availability of energy substrates to fuel the mitochondria, and the joint dynamics of mitochondrial ‘fusion and fission’ [126,127]. Fission and fusion are coordinated by a family of large GTPases and their respective adaptor proteins. The correct balance between mitochondrial fission and fusion is critical to the maintenance of healthy cellular homeostasis. Any misalignment in the balance of fission and fusion is detrimental to the cell and widely linked to neurodegenerative progression.

## 5. Microglia Activation and Neurodegeneration in Ageing and Parkinson’s Disease

Microglia are the resident immune cells of the brain interacting with astrocytes [128], oligodendrocytes, neurons, and brain vasculature via autocrine (non-local) and paracrine (local) cell signalling [129,130] to maintain homeostatic regulation of neuronal homeostatic processes [6,124,131], blood brain barrier (BBB) integrity [132,133], synaptic pruning [134], synaptogenesis [96], and neurogenesis [135]. Microglial dysregulation has also been widely identified as a major contributor to elevated levels of neurotoxins and ongoing chronic activation of microglia associated with neuroinflammatory damage [136,137,138], protein misfolding [139], and neurodegenerative progression in PD [140,141]. Postmortem research [142] and meta-analytic results [143] have confirmed significantly higher microglial activation and concentrations of common inflammatory cytokines, IL-6, TNF, IL-1β, IL-2, IL-10, and C-reactive protein, in ageing and in the SNpC of PD patients compared to healthy controls (HC) of the same age [3,73,130,144,145]. Ouchi et al. [142] also observed that microglial activation in PD was significantly elevated above what could be explained by age-related microglial-driven inflammation in PD models, while Zhang et al. [78] have found increased numbers of reactive microglia that were well correlated with declining density and overall numbers of dopaminergic neurons in the SNpC of PD patients. Furthermore, microglial activation in the LC has also been found to trigger elevated production of vascular (VCAM-1) and intracellular (ICAM-1) cell adhesion molecules linked with increased dopaminergic neuronal apoptosis in PD progression [146].

Preservation of dopaminergic neurons in patients diagnosed with PD requires efficient anti-inflammatory mechanisms to inhibit chronic neuroinflammation and maintain cellular homeostasis [147]. Indeed, pre-clinical PD studies, reviewed elsewhere (146), have found that dysregulation of anti-inflammatory agents such as polyunsaturated fatty acids (PUFA) (147) can cross the blood brain barrier (BBB) and inhibit microglial activation to help lower inflammatory neuronal damage in PD [148]. Upregulation of activated proinflammatory (M1) microglia due to increased dopaminergic neuronal apoptosis in PD progression [146] has also been associated with increased release of ROS from damaged mitochondria [49,82], increased misfolded protein aggregates, such as α-Syn within Lewy bodies, and autophagic mechanisms leading to elevated neurotoxicity [149,150,151,152,153,154]. The elevated levels of ROS cause overactivation of the intracellular protein Nucleotide Oligomerization Domain (NOD), which plays a critical role in triggering a microglial-mediated proinflammatory response [73,155]. Increased ROS and NOD activation also affects the regulation of the innate immune response, stimulating the NLRP3 receptor for inflammasome-dependent inflammatory pathways that are involved in triggering a microglial-mediated proinflammatory response [73,139]. George, et al. [156] also noted that misfolded α-Syn protein exhibited paracrine properties, moving from cell to cell [150], promoting further α-Syn aggregation, and potentially acting as a chemoattractant to microglial cells, contributing to microglial aggregation, accelerated neuroinflammation, and increased cell death of dopaminergic neurons in most types of neurodegenerative diseases, including PD [157].

**Figure 3:**
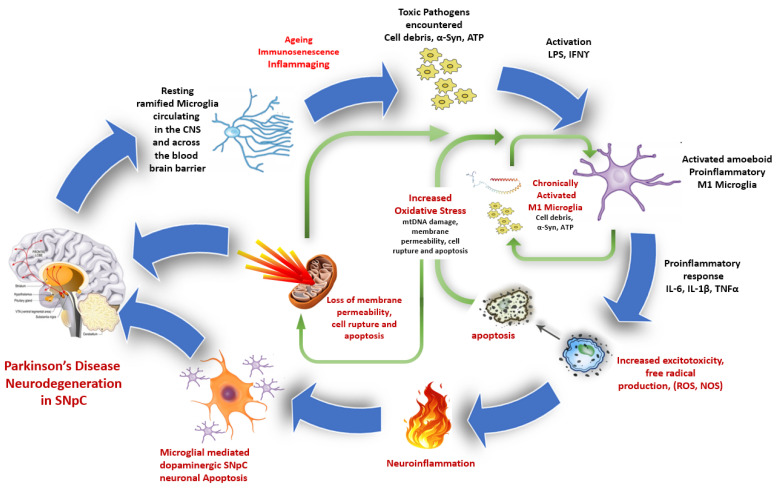
Microglial-mediated proinflammatory SNpC neuronal damage. Normally, macrophages circulate in the blood system throughout the body, i.e., peripherally and centrally, in a resting quiescent state. However, ageing, linked to mild but persistent inflammation, means microglia of the CNS can become increasingly and chronically activated at a faster rate when compared to healthy controls. Pathogens such as excitotoxins, protein aggregates like α-Syn, and apoptotic cell debris, such as ATP and mitochondrial (mt)DNA released into the cytosol from dying mitochondria and cells, can act as transient initiators, triggering and perpetuating an escalating immune response [167,168]. As part of the initial immune response, proinflammatory M1 microglia release cytokines to remove anomalous ions such as excessive sodium or potassium induced by hypertension [169] or detected pathogens [170,171]. These pathogens exacerbate the level of toxicity, causing further elevation in microglial activation and increasing excitotoxicity, free radical production, and neuroinflammation. These changes begin to create a damaging feedback loop, activating higher numbers of microglia for longer and moving them towards potentially chronic levels. Associated with this bio-feedback loop, increases in levels of chronic microglial activation have been linked to greater energy needs, loss of mitochondrial membrane permeability, increased oxidative stress, and microglial-mediated dopaminergic apoptosis, promoting further elevated innate immune responses in the SNpC of PD patients [172].

While proinflammatory cytokine release is a core part of the protective immune response, as more research is undertaken on microglial activation scenarios [158], a polarised categorisation of either a proinflammatory M1 or anti-inflammatory M2 phenotype is beginning to seem too restrictive [159]. Single-cell RNA (scRNA) sequencing techniques are now showing a broad range of specialised microglia clusters [160] that change during development [159,161] and respond to differing environmental stressors, such as cellular damage from bacteria and viruses, accumulating cellular debris, and sensing and triggering mechanisms, such as pathogen and damage-associated molecular patterns (PAMPs and DAMPs). Such specialised microglial clusters may suggest that these different types of microglia activate along a continuum [3], with different levels of response commensurate with specific stimuli [162,163,164,165]. More recent evidence presented by Gertig et al. [166] using stimulated mouse microglia revealed clear sub-populations of microglia possessing different functional attributes and signalling properties that became more distinct with increasing ageing. Thus, a broader definition of microglial activation state incorporates ideas of activation phenotypes encompassing early defensive action to detect, locate, and remove pathogens encountered through to later actions that include tissue repair and restoration of cellular structural integrity [166]. Conceptually, microglial activation along a continuum in response to a variety of specific and different stimuli of varying time and influences is consistent with a progressive and escalating pattern of microglial mediator-driven responses associated with progressive neurodegenerative disease. See Figure 3 below.

Although microglia are present in large numbers throughout the brain, they are not uniformly distributed or activated across all nuclei [160,173], with density being triggered by different stimuli [162,163,165,166]. Proinflammatory M1 and anti-inflammatory M2 microglia co-exist in the SNpC of PD brains, but in terms of microglial pro/anti-inflammatory balance, it has been reported that there is a greater volume of inflammatory phenotypes compared to anti-inflammatory phenotype numbers in the SNpC of PD patients [17,174]. Thus, the elevated abundance of activated M1 compared to the anti-inflammatory M2 microglia would be expected to lead to greater neurodegenerative damage in SNpC compared to other areas of the brain [131,141].

Postmortem analysis of microglial distribution taken from normal, healthy adult mice has shown around a fivefold difference in number and heterogeneous distribution in the density of microglia between brain regions [173]. Substantially higher numbers of microglia have been reported in the hippocampus, olfactory telencephalon, basal ganglia, and SNpC, suggesting these areas of the brain are possibly more prone to ageing [17,27,175] and earlier neuroinflammatory damage [17,173] compared to other brain areas. Furthermore, higher concentrations of activated microglia in non-posterior areas of the SNpC, particularly when exacerbated by ageing, have been associated with an elevated, continuous, and excessive production of proinflammatory M1 microglial mediators, including IL-1β, IL-6, IL-8, IL-12, and TNF-α [176,177]. The elevated number of activated microglia was found to be toxic to neurons [178,179,180,181], promoting significantly higher levels of neurodegenerative damage and apoptosis in the disease progression of PD [168,182]. By comparison, animal postmortem results using immunohistochemistry of other non-SNpC brain areas, such as fibre tracts, cerebellum, and brainstem, taken from the same euthanised mouse [173], showed much lower microglial densities, with commensurately lower levels of apoptosis and functional loss following normal microglial proinflammatory activation [3,168,183].

Gene susceptibility has been investigated in PD with a number of early human epidemiological studies and meta-analyses investigating the role of Apolipoprotein E (*ApoE*) and cholesterol metabolism in PD pathogenesis [184,185,186], following the discovery that carriers of the *ApoE*-ε4 isoform gene showed a higher probability of progression onto PD, while those with *ApoE*-ε2 isoform showed a level of protection against developing PD [184,187,188]. *ApoE* is a ubiquitous plasma protein synthesised in the human liver and brain by glia, macrophages, and neurons [188,189]. *APOE* plays a key role in the metabolism of fats, with cholesterol being a major component of synapses and cell membranes essential for maintaining the functioning and structural integrity of the neurons [186]. Interestingly, the *APOE ε4* isoform gene has been linked to more severe and faster rates of cognitive decline in both PD mouse models and human studies [190,191]. Furthermore, it has been found using *APOE ε4* mouse models that the number of hippocampal neurons containing high levels of upregulated *APOE* expression, increases dramatically prior to clinical diagnosis and the onset of neurodegenerative symptomology, then declines swiftly at the onset of the pathology as neurodegenerative impairment and cognitive decline become more apparent [192,193], suggesting high levels of *APOE* are implicated in the early onset of PD and cognitive loss but potentially less of an influence in the later stages ongoing stage of neurodegenerative and cognitive damage [192]. PD research using *ApoE* mice has also linked increased levels of *ApoE* to higher neuronal stress and activation of microglia, triggering the release of the cytokine interferon-γ (*IF-γ*) and upregulation of neuronal major histocompatibility complex class I (MHC-I) levels [192], which has the effect of making the neurons more easily recognised and destroyed by T cells [192,194,195]. Several studies have also linked the presence of the *APOEε4* isoform gene to reduced mitochondrial fission and impaired mitophagy under both basal and oxidative stress situations [196,197,198,199], though the mechanisms and dynamics of such an APOEε4 isoform gene-associated impaired mitochondrial function remain poorly understood and controversial [197,200].

## 6. Influence of Ageing on Microglia and Mitochondria in PD Neurodegeneration

### 6.1. Age as the Key Risk Factor of PD

**Figure 4:**
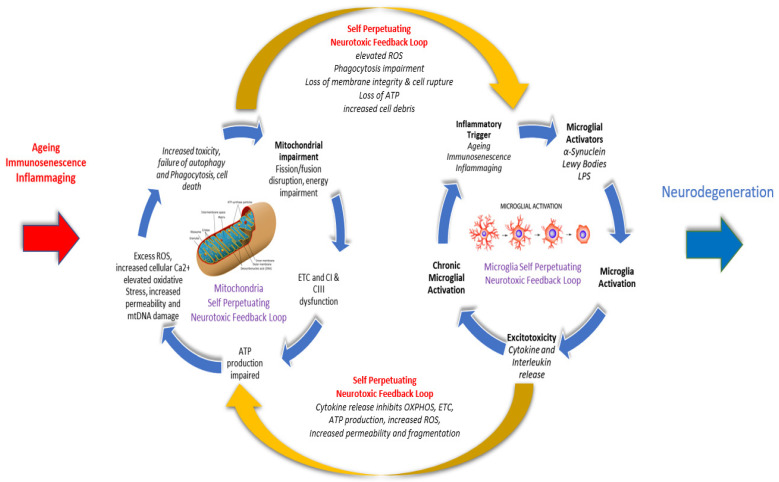
Self-perpetuating bi-directional neurotoxic feedback loops between mitochondrial impairment and microglial over-activation. Bi-directional communications between neuronal mitochondria and microglia are part of the cellular innate immune response and are implicated in central neural inflammation and the loss of dopaminergic neurons, leading to neurodegenerative progression in the LC and SNpC of Parkinson’s disease patients. Mitochondrial impairment as a result of electron transport chain dysfunction, lower ATP output, elevated levels of ROS, oxidative stress, increased mitochondrial membrane permeability, failure of membrane structure, and failure of phagocytotic clearance each contribute to increased cellular toxicity and apoptosis. Failure of membrane structure and phagocytotic clearance then triggers a microglial-mediated immune response to mitigate escalating inflammation because of increasing cytotoxicity. As the neuron attempts to manage increasing inflammatory damage and cytokine release, it also results in further neuronal mitochondrial impairment, triggering rising microglial activation to chronic levels, perpetuating the neuronal damage, cognitive impairment, and motor dysfunction [215,216].

While age is the key risk factor for PD [201,202,203,204], neurodegenerative progression of PD has long been associated with escalating neuroinflammation from chronic immunological activation of the microglial system in the brain [132,137,168,172,205], but seldom considered in terms of the concurrent effects of ageing on neurons for which substantial evidence now exists. Indeed, reduced mitochondrial membrane integrity and degradation of the energetic function of neuronal mitochondria are inevitably linked with ageing [92,110,204,206,207,208] and associated with the innate immune system responses alluded to previously. These responses include damage-induced microglial activation [8,38,112,119,209,210,211,212,213], signalling interactions [200] with the vascular system, and self-perpetuating feedback loops that further exacerbate neuroinflammation and escalate neurodegenerative damage in PD [119,214]. See Figure 4.

### 6.2. Inflammation and Inflammaging in Ageing

Ageing and age-related diseases such as PD share a number of basic mechanistic pillars that converge on inflammation after the age of 40 [25]. During ageing, there is overwhelming epidemiological, biological, and metabolic evidence of age-acquired immuno-dysfunction and increasing microglial density across different areas of the brain over the lifespan [217,218,219]. Additionally, these changes are combined with an ongoing and escalating chronic, low-grade inflammation, called inflammaging [23,215], taking place without evidence of primary infection [220]. Beyond 40 years of age [25], immunosenescence is responsible for the gradual and consistent rise in cumulative low-level inflammation [24,145], with a commensurate deterioration of the innate immune system’s capacity to respond efficiently to pathogenic removal [24], downregulation in the autophagic expression associated with compromised phagocytosis [201], and the management and control of cellular homeostasis [26]. The decreased activity in phagocytosis during ageing [221] further compromises the microglia’s ability to effectively clear toxic substances from the cell and restore homeostasis [222]. Impaired phagocytosis then leads to increased neurotoxicity and cellular senescence in response to cellular damage and stress, increasing oxidative stress and ROS, and increasing cellular death [6,223,224,225].

Impaired activation of the adult immune system during ageing has also been linked to the pathogenesis of obesity-related insulin resistance [226,227], type 2 diabetes [228,229] and increased risk of autoimmune disorders, such as rheumatoid arthritis [202,230], and vascular conditions, including hypertension (high BP) and hypotension (low BP) [231]. These chronic conditions are also linked to age-driven inflammaging, with its increased risk of micro- and macrovascular complications, such as persistent blood pressure anomalies and small blood vessel disorders, which contribute to age-related neurodegenerative disorders such as PD [145]. Interestingly, such age related chronic medical conditions have also been reported to show evidence of mitochondrial disease [111,232,233,234,235,236,237,238,239], including inadequate ATP availability, dysfunction in regulating cytoplasmic and mitochondrial calcium levels, and mitophagy linked to rising toxic levels of ROS [240]. Thus, it is no surprise that medical comorbidities such as orthostatic hypertension/hypertension, olfactory loss, sleep disruption, persistent constipation, and diabetes are highly correlated in predicting PD and the progression of the disease [228,241,242].

### 6.3. Mitochondrial Dysfunction and PD

As alluded to earlier and shown in Figure 3 and Figure 4, impaired or damaged neurons in the SNpC show mitochondrial dysfunction in terms of increased mutations in mtDNA and mitochondrial ribonucleic acid (mtRNA), loss of ETC efficiency, deficiencies in ATP production [243,244,245], increased proton leakage [118,246], and increasing production of neurotoxic free radicals, such as intracellular nitrous oxide synthase (NOS) [131], ROS [114,122,124,247,248,249,250], and increased release of ATP and mitochondrial debris into the cytosol [125,251], see Figure 2. These molecules interact with the same specialised cytoplasmic sensors that detect cellular pathogens, triggering a microglial-mediated inflammatory immune response [252] within the SNpC, leading to apoptosis and the loss of dopaminergic neurons. The release of mtDNA, mtRNA, and ATP into the cytosol also activates various immune signalling receptors, including Toll Like Receptor (TLR9), Nod Like Receptor (NLRP3), and Stimulator of interferon genes (STING), which are involved in facilitating and regulating antibacterial and antiviral immunity as part of the proinflammatory immune response [253]. In addition, the loss of cellular energetics has been linked to lower immune system efficiency, resulting in reductions in natural killer (NK) cells, total CD8+ T cells, and CD8+ memory T cells associated with evidence of higher viral and bacterial infection being revealed in patient case histories [110,254]. Similar results have been reported when using immortalised lymphocytes isolated from idiopathic PD patients [123,255]. Mitochondrial mass, genome copy number, and membrane potential in the lymphoblasts were found to be functionally normal but hyperactive and producing significantly elevated levels of neurotoxic damaging ROS in PD patients compared to healthy controls.

Dopaminergic axons in the SNpC have one of the largest energetic demands in the body; however, they also have limited surplus or reserve energetic capacity [256]. In order to maintain efficient basal metabolic functioning [257,258], synaptic transmissions, and cell survival [84], dopaminergic neurons require consistent and regular supplies of mitochondria and ATP, which are met by the neuron maintaining high concentrations of mitochondria at the axon terminal. Any interruption or insufficient energetic availability can be catastrophic for the cell, resulting in significant cellular damage, apoptosis, and progressive neuronal degeneration in PD [251]. In mature neurons and presynaptic structures, around 87% of mitochondria are stationary, residing in a highly structured reticular state [259], and need to be transported along axons in the neuron to areas of high energetic demand. Any disruption to the movement of mitochondria along the axon via microtubule-based transport and anchoring substrates [259] can be damaging to mitochondrial redistribution to manage physiological or pathological cellular stress in order to maintain energic homeostasis [125,260]. Research findings by Ray, et al. [261] have found disruption of mitochondrial crosstalk between organelles, including endoplasmic reticulum (ER), peroxisomes, and lysosomes, triggered significant changes in intracellular vesicle transporters and calcium buffering, which were implicated with increased protein misfolding, protease activation, and impaired autophagic clearance, all of which are key processes contributing to neurodegenerative progression in PD [112].

Mitochondrial damage has critical implications for the maximum ATP that can be produced and made available to the cell under pathogenic stress to maintain cellular homeostasis and interactions with other cellular and vascular innate immune processes [262]. Given the mitochondria’s low energy storage capacity and minimal surplus ATP availability, any interruption or deficiencies in the energy supply to the axons will impair or halt energy-driven neuronal events, leading to irreversible dopaminergic loss and neurodegeneration in the SNpC [263,264]. Functional magnetic resonance imaging (fMRI), magnetic resonance spectroscopy (MRS), and positron emission tomography (PET) studies support these findings, showing that PD patients achieve significantly lower brain glucose utilisation, suggesting lower ATP production and availability in the brain regions most affected by energetic loss, such as the SNpC in PD progression [61]. The lack of brain glucose uptake is also directly linked with subsequent cognitive decline in neurodegenerative diseases such as PD [265].

The evidence presented here demonstrates strong causal associations between mitochondrial dysfunction and impaired ETC efficiency, resulting in excess production of neurotoxic ROS and inducible nitric oxide synthase (iNOS), microglial-mediated proinflammatory cytokine release, neuroinflammation, and progressive neuronal cell death. These relationships are continuously and dynamically changing in response to the cellular environment, resulting in concurrent bidirectional associations in key signalling and molecular mechanisms linked to diminished ATP availability and impaired neuronal homeostasis, with direct consequences linked to activation of microglia and progressive neurodegeneration in PD. As a consequence of the inter-related roles that mitochondria perform in the cell, any impairment or dysfunction can often appear early in neurodegenerative progression, potentially offering a metabolic target to assist in the prodromal diagnosis of PD [263].

## 7. Limitations

The greatest limitation associated with much of the experimental evidence reviewed here is that it is either based on static postmortem analysis or in vivo/in vitro animal studies with a specific and often narrow molecular and time focus combined with a restrictive young age of the animals used in the experiments. As a result, much of the research reviewed does not fully represent the multidimensional dynamics, spontaneity, and inter-related biological relationships between the reciprocal feedback signalling loops involved in proinflammatory chronic microglia activation and mitochondrial energetic impairment in aged humans. While in vivo animal studies may reflect some of the higher-order complex signalling and biological relationships evident in humans, experimental in vitro data often reflects specific laboratory-based culture media that have usually been drawn from younger animal sources rather than from aged sick animal. As a result, experiments mostly focus on a single stream of cause-and-effect relationships rather than on more complex systems of multiple signalling cascades as inputs and outputs. As an example, past in vitro studies have suggested increased levels of neurotoxicity resulting from the oxidation of dopamine being linked to mitochondrial dysfunction, protein degradation, and elevated ROS production [266]. However, such results are particularly difficult to correlate with the impact of dopaminergic oxidation across differing areas of the human brain [81] which makes the findings difficult to use in identifying new metabolic, pharmacological and/or molecular signalling targets as the basis of successful clinical treatments in humans. Experiments using aged mice and primates treated with MPTP have clearly shown dopaminergic neuronal loss and the pathological features of PD; however, these models failed to also show Lewy body aggregation and the prion-type spreading impacts of α-Syn, which is now acknowledged as a core characteristic of neurodegeneration in PD [27,267] that occurs as the disease progresses [268].

Furthermore, it can be difficult to obtain sufficient human postmortem PD brain tissue samples to reach statistically significant and consistent results across comparative experimental designs due to the diversity in each human sample [75,128], leaving a heavy reliance on animal models for reliability and validity. While animal model data can be genetically consistent, experimental results continue to be difficult to translate from young animal studies to elderly human neurodegenerative PD patients [269]. The issue of age in experimentation also becomes problematic when attempting to apply animal findings across human clinical and medical trials. In these cases, targeted pharmaceutical compounds often have differing outcomes and side effects in animals [270] compared to humans, lowering the efficacy of the results and cost effectiveness for human clinical treatment success.

A further problem encountered is that neurodegenerative disease progression is an age-related and time-dependent process with incremental neuronal damage resulting from prolonged and elevated proinflammatory responses associated with excitotoxicity, protein aggregates and mutations, constant low-grade inflammation “inflammaging” [26], mitochondrial dysfunction, cellular ageing, and immunosenescence accumulating over time for up to 15 years prior to clinical diagnosis [25]. Evidence of age-related neuronal damage from activated microglia has been found in human postmortem PD brains up to 16 years after initial cytotoxic exposure triggered the initial proinflammatory response [271]. Therefore, the use of longitudinal data in neurodegenerative research for both PD patients and healthy controls may assist in the analysis and interpretation of longer-term relationships between cell signalling impacts and age-related immunosenescence combined with a prolonged exposure of the cell to insufficient or low mitochondrial energetics. However, the sheer length and ongoing prohibitive costs of any longitudinal study to mimic a lengthy prodromal period of up to 15–20 years [10] may also make evaluation of experimental data difficult. Such evaluations would also be exacerbated by the accumulation of simultaneously developing confounding factors associated with ageing and comorbid diseases associated with the onset and progression of PD [241].

## 8. Conclusions

Parkinson’s disease is a chronic and progressive age-related neurodegenerative disease [4], clinically diagnosed with the emergence of cognitive, behavioural, and key motor deficits [28,64,272,273]. Dopaminergic neurons in the SNpC that are highly energy dependent with limited surplus or reserve energetic capacity [257,258] are often seen as causative in PD. However, the caudo-rostral progression of the disease and many other studies cited in this review note early impairments to the autonomic immune system and locus coeruleus functions, e.g., sleep-wake routines, cognitive and affective characteristics, and orthostatic blood pressure, many years prior to clinical PD diagnosis of motor symptoms, making this a challenging issue to both understand and progress towards a solution. Furthermore, age-related inflammaging causing interruptions in the ATP supply to the neurons can be highly damaging, leading to irreversible dopaminergic loss and neurodegeneration, particularly in the SNpC in PD [263,264].

Emerging evidence of metabolic pathway similarities and the existence of crosstalk between microglia and mitochondria has been confirmed by findings that the signalling node that regulates the microglial transition to a proinflammatory state utilises the same pathway involved in switching mitochondrial ATP production from OXPHOS to glycolysis in the cytosol [165,274,275]. It has also been shown that excess mitochondrial ROS inhibits ETC efficiency at complex I, causing a significant decrease in mitochondrial oxygen consumption consistent with lower ATP production, simultaneously transforming microglia into a more severe neurotoxic phenotype and inducing a further increase in free radical production, such as nitric oxide. These changes are associated with chronic microglial activation and cytokine secretion [276], which is reported to be up to 70 times more active in the SNpC of PD patients compared to normal HCs [46,277].

This review suggests there is strong evidence for investigating PD progression and neurodegenerative damage as multifaceted health impairments. Such changes need to be considered in association with ageing and influenced by continuous and concurrent bidirectional signalling and interrelated crosstalk between impaired neuronal mitochondrial function and cellular dysfunction, resulting in increased numbers of chronically activated microglia. Alterations in signalling pathways need to be considered dynamic and bidirectional rather than independent and linear at a static point in time. Mitochondrial dysfunction resulting in elevated oxidative stress and ROS, impaired ATP energy production, and increased cellular debris released into the cytosol and extracellular matrix, together with age and sleep-disturbance related glymphatic drainage, lead to an escalation in microglial-mediated proinflammatory immune responses to chronic levels [278]. Concurrently, the independent impact of ageing and general inflammatory/inflammaging responses trigger ongoing and escalating proinflammatory brain-based microglial-mediated immune responses. These changes increase misfolded protein aggregates, such as α-Syn within Lewy bodies, which further damage neuronal mitochondria membrane integrity and autophagic impairment, leading to elevated neurotoxicity from increased release of ROS, which then stimulates further microglial inflammatory responses. A better understanding of the molecular mechanisms driving these reciprocal feedback loops may assist in identifying high-probability pharmacological targets of neurodegenerative disease and have future clinical implications for slowing or stopping further disease progression early in the lengthy prodromal stages of PD.

## References

[1] Kim S.D., Allen N.E., Canning C.G., Fung V.S.C. (2018). Parkinson Disease.

[2] Johnson M.E., Bobrovskaya L. (2015). An update on the rotenone models of Parkinson’s disease: Their ability to reproduce the features of clinical disease and model gene-environment interactions. Neurotoxicology.

[3] Tansey M.G., Wallings R.L., Houser M.C., Herrick M.K., Keating C.E., Joers V. (2022). Inflammation and immune dysfunction in Parkinson disease. Nat. Rev. Immunol..

[4] Boyko A.A., Troyanova N.I., Kovalenko E.I., Sapozhnikov A.M. (2017). Similarity and Differences in Inflammation-Related Characteristics of the Peripheral Immune System of Patients with Parkinson’s and Alzheimer’s Diseases. Int. J. Mol. Sci..

[5] Deumens R., Blokland A., Prickaerts J. (2002). Modeling Parkinson’s disease in rats: An evaluation of 6-OHDA lesions of the nigrostriatal pathway. Exp. Neurol..

[6] Tremblay M.E., Cookson M.R., Civiero L. (2019). Glial phagocytic clearance in Parkinson’s disease. Mol. Neurodegener..

[7] Picca A., Guerra F., Calvani R., Bucci C., Lo Monaco M.R., Bentivoglio A.R., Landi F., Bernabei R., Marzetti E. (2019). Mitochondrial-Derived Vesicles as Candidate Biomarkers in Parkinson’s Disease: Rationale, Design and Methods of the EXosomes in PArkiNson Disease (EXPAND) Study. Int. J. Mol. Sci..

[8] Koziorowski D., Figura M., Milanowski Ł.M., Szlufik S., Alster P., Madetko N., Friedman A. (2021). Mechanisms of Neurodegeneration in Various Forms of Parkinsonism, Similarities and Differences. Cells.

[9] Cooper C.A., Chahine L.M. (2016). Biomarkers in Prodromal Parkinson Disease: A Qualitative Review. J. Int. Neuropsychol. Soc..

[10] Gaig C., Tolosa E. (2009). When does Parkinson’s disease begin?. Mov. Disord..

[11] Berg D., Borghammer P., Fereshtehnejad S.M., Heinzel S., Horsager J., Schaeffer E., Postuma R.B. (2021). Prodromal Parkinson disease subtypes—Key to understanding heterogeneity. Nat. Rev. Neurol..

[12] Muslimovic D., Post B., Speelman J.D., De Haan R.J., Schmand B. (2009). Cognitive decline in Parkinson’s disease: A prospective longitudinal study. J. Int. Neuropsychol. Soc..

[13] Zhou Z., Zhou X., Zhou X., Xiang Y., Zhu L., Qin L., Wang Y., Pan H., Zhao Y., Sun Q. (2021). Characteristics of Autonomic Dysfunction in Parkinson’s Disease: A Large Chinese Multicenter Cohort Study. Front. Aging Neurosci..

[14] Shin J.H., Lee J.Y., Kim Y.K., Yoon E.J., Kim H., Nam H., Jeon B. (2021). Parkinson Disease-Related Brain Metabolic Patterns and Neurodegeneration in Isolated REM Sleep Behavior Disorder. Neurology.

[15] Stokholm M.G., Iranzo A., Ostergaard K., Serradell M., Otto M., Svendsen K.B., Garrido A., Vilas D., Borghammer P., Santamaria J. (2017). Assessment of neuroinflammation in patients with idiopathic rapid-eye-movement sleep behaviour disorder: A case-control study. Lancet Neurol..

[16] Schrag A., Sauerbier A., Chaudhuri K.R. (2015). New clinical trials for non-motor manifestations of Parkinson’s disease. Mov. Disord..

[17] Bachiller S., Jimenez-Ferrer I., Paulus A., Yang Y., Swanberg M., Deierborg T., Boza-Serrano A. (2018). Microglia in Neurological Diseases: A Road Map to Brain-Disease Dependent-Inflammatory Response. Front. Cell. Neurosci..

[18] Schrag A., Horsfall L., Walters K., Noyce A., Petersen I. (2015). Prediagnostic presentations of Parkinson’s disease in primary care: A case-control study. Lancet Neurol..

[19] Cardoso S.M., Empadinhas N. (2018). The Microbiome-Mitochondria Dance in Prodromal Parkinson’s Disease. Front. Physiol..

[20] Dogra N., Mani R.J., Katare D.P. (2022). The Gut-Brain Axis: Two Ways Signaling in Parkinson’s Disease. Cell. Mol. Neurobiol..

[21] Fearnley J.M., Lees A.J. (1991). Ageing and Parkinson’s disease: Substantia nigra regional selectivity. Brain.

[22] Moca E.N., Lecca D., Hope K.T., Etienne F., Schaler A.W., Espinoza K., Chappell M.S., Gray D.T., Tweedie D., Sidhu S. (2022). Microglia Drive Pockets of Neuroinflammation in Middle Age. J. Neurosci..

[23] Franceschi C., Bonafe M., Valensin S., Olivieri F., De Luca M., Ottaviani E., De Benedictis G. (2000). Inflammaging. An evolutionary perspective on immunosenescence. Ann. N. Y. Acad. Sci..

[24] Beltran-Castillo S., Eugenin J., von Bernhardi R. (2018). Impact of Aging in Microglia-Mediated D-Serine Balance in the CNS. Mediat. Inflamm..

[25] Tangestani F.M., Stough C. (2019). A Review and Hypothesized Model of the Mechanisms That Underpin the Relationship between Inflammation and Cognition in the Elderly. Front. Aging Neurosci..

[26] Batatinha H.A.P., Diniz T.A., de Souza Teixeira A.A., Kruger K., Rosa-Neto J.C. (2019). Regulation of autophagy as a therapy for immunosenescence-driven cancer and neurodegenerative diseases: The role of exercise. J. Cell. Physiol..

[27] Hou Y., Dan X., Babbar M., Wei Y., Hasselbalch S.G., Croteau D.L., Bohr V.A. (2019). Ageing as a risk factor for neurodegenerative disease. Nat. Rev. Neurol..

[28] Galtier I., Nieto A., Lorenzo J.N., Barroso J. (2016). Mild cognitive impairment in Parkinson’s disease: Diagnosis and progression to dementia. J. Clin. Exp. Neuropsychol..

[29] Safarpour D., Willis A.W. (2016). Clinical Epidemiology, Evaluation, and Management of Dementia in Parkinson Disease. Am. J. Alzheimers Dis. Other Dement..

[30] Kalia L.V., Lang A.E. (2015). Parkinson’s disease. Lancet.

[31] Urbani A., Babu M. (2019). Mitochondria in Health and in Sickness.

[32] Larsson N.G., Wedell A. (2020). Mitochondria in human disease. J. Intern. Med..

[33] Duchen M.R., Szabadkai G. (2010). Roles of mitochondria in human disease. Essays Biochem..

[34] Khacho M., Clark A., Svoboda D.S., MacLaurin J.G., Lagace D.C., Park D.S., Slack R.S. (2017). Mitochondrial dysfunction underlies cognitive defects as a result of neural stem cell depletion and impaired neurogenesis. Hum. Mol. Genet..

[35] Las G., Oliveira M.F., Shirihai O.S. (2020). Emerging roles of beta-cell mitochondria in type-2-diabetes. Mol. Asp. Med..

[36] Bonora M., Wieckowski M.R., Sinclair D.A., Kroemer G., Pinton P., Galluzzi L. (2019). Targeting mitochondria for cardiovascular disorders: Therapeutic potential and obstacles. Nat. Rev. Cardiol..

[37] Forte M., Palmerio S., Bianchi F., Volpe M., Rubattu S. (2019). Mitochondrial complex I deficiency and cardiovascular diseases: Current evidence and future directions. J. Mol. Med..

[38] Giummarra L., Crewther S.G., Riddell N., Murphy M.J., Crewther D.P. (2018). Pathway analysis identifies altered mitochondrial metabolism, neurotransmission, structural pathways and complement cascade in retina/RPE/choroid in chick model of form-deprivation myopia. PeerJ.

[39] Dorsey E.R., Sherer T., Okun M.S., Bloem B.R. (2018). The Emerging Evidence of the Parkinson Pandemic. J. Park. Dis..

[40] Evans A.H. (2020). Parkinson’s during COVID-19: Then, Now and Next. Parkinson’s during COVID-19: Managing Treatments and Stress during a Pandemic.

[41] Marchetti B., Giachino C., Tirolo C., Serapide M.F. (2022). “Reframing” dopamine signaling at the intersection of glial networks in the aged Parkinsonian brain as innate Nrf2/Wnt driver: Therapeutical implications. Aging Cell.

[42] Pezzoli G., Cereda E. (2013). Exposure to pesticides or solvents and risk of Parkinson disease. Neurology.

[43] Weisskopf M.G., Weuve J., Nie H., Saint-Hilaire M.H., Sudarsky L., Simon D.K., Hersh B., Schwartz J., Wright R.O., Hu H. (2010). Association of cumulative lead exposure with Parkinson’s disease. Environ. Health Perspect..

[44] Cova I., Markova A., Campini I., Grande G., Mariani C., Pomati S. (2017). Worldwide trends in the prevalence of dementia. J. Neurol. Sci..

[45] Badanjak K., Fixemer S., Smajic S., Skupin A., Grunewald A. (2021). The Contribution of Microglia to Neuroinflammation in Parkinson’s Disease. Int. J. Mol. Sci..

[46] Suescun J., Chandra S., Schiess M.C. (2019). The Role of Neuroinflammation in Neurodegenerative Disorders. Translational Inflammation.

[47] Kasap M., Akpinar G., Kanli A. (2017). Proteomic studies associated with Parkinson’s disease. Expert Rev. Proteom..

[48] Rocca W.A. (2018). The burden of Parkinson’s disease: A worldwide perspective. Lancet Neurol..

[49] Hirsch L., Jette N., Frolkis A., Steeves T., Pringsheim T. (2016). The Incidence of Parkinson’s Disease: A Systematic Review and Meta-Analysis. Neuroepidemiology.

[50] Bhimani R. (2014). Understanding the Burden on Caregivers of People with Parkinson’s: A Scoping Review of the Literature. Rehabil. Res. Pract..

[51] Dorsey E.R., Bloem B.R. (2018). The Parkinson Pandemic—A Call to Action. JAMA Neurol..

[52] Dorsey E.R., Constantinescu R., Thompson J.P., Biglan K.M., Holloway R.G., Kieburtz K., Marshall F.J., Ravina B.M., Schifitto G., Siderowf A. (2007). Projected number of people with Parkinson disease in the most populous nations, 2005 through 2030. Neurology.

[53] Dorsey E., Elbaz A., Nichols E., Abd-Allah F., Abdelalim A., Adsuar J., Ansha M., Brayne C., Choi J.-Y., Collado-Mateo D. (2018). Global, regional, and national burden of Parkinson’s disease, 1990–2016: A systematic analysis for the Global Burden of Disease Study 2016. Lancet Neurol..

[54] Schulz R., Beach S. (1999). Caregiving as a risk factor for mortality: The Caregiver Health Effects Study. JAMA.

[55] Beauchamp L.C., Finkelstein D.I., Bush A.I., Evans A.H., Barnham K.J. (2020). Parkinsonism as a Third Wave of the COVID-19 Pandemic?. J. Park. Dis..

[56] Helmich R.C., Bloem B.R. (2020). The Impact of the COVID-19 Pandemic on Parkinson’s Disease: Hidden Sorrows and Emerging Opportunities. J. Park. Dis..

[57] Wijeratne T., Crewther S. (2020). Post-COVID 19 Neurological Syndrome (PCNS); a novel syndrome with challenges for the global neurology community. J. Neurol. Sci..

[58] Delenclos M., Jones D.R., McLean P.J., Uitti R.J. (2016). Biomarkers in Parkinson’s disease: Advances and strategies. Park. Relat. Disord..

[59] Dauer W., Przedborski S. (2003). Parkinson’s disease: Mechanisms and models. Neuron.

[60] Jankovic J. (2019). Pathogenesis-targeted therapeutic strategies in Parkinson’s disease. Mov. Disord..

[61] Camandola S., Mattson M.P. (2017). Brain metabolism in health, aging, and neurodegeneration. EMBO J..

[62] Stojkovic T., Stefanova E., Soldatovic I., Markovic V., Stankovic I., Petrovic I., Agosta F., Galantucci S., Filippi M., Kostic V. (2018). Exploring the relationship between motor impairment, vascular burden and cognition in Parkinson’s disease. J. Neurol..

[63] Kim I., Shin N.Y., Yunjin B., Hyu Lee P., Lee S.K., Mee Lim S. (2017). Early-onset mild cognitive impairment in Parkinson’s disease: Altered corticopetal cholinergic network. Science.

[64] Galtier I., Nieto A., Barroso J., Dorszewska J. (2016). Cognitive Impairment in Parkinson’s Disease: Historical Review, Past, and Present.

[65] Braak H., Ghebremedhin E., Rub U., Bratzke H., Del Tredici K. (2004). Stages in the development of Parkinson’s disease-related pathology. Cell Tissue Res..

[66] Zarow C., Lyness S., Mortimer J., Chui H. (2003). Neuronal loss is greater in the locus coeruleus than nucleus basalis and substantia nigra in Alzheimer and Parkinson diseases. Arch. Neurol..

[67] Del Tredici K., Rüb U., De Vos R., Bohl J., Braak H. (2002). Where does Parkinson disease pathology begin in the brain?. J. Neuropathol. Exp. Neurol..

[68] Yao N., Wu Y., Zhou Y., Ju L., Liu Y., Ju R., Duan D., Xu Q. (2015). Lesion of the locus coeruleus aggravates dopaminergic neuron degeneration by modulating microglial function in mouse models of Parkinson’s disease. Brain Res..

[69] Fornai F., di Poggio A.B., Pellegrini A., Ruggieri S., Paparelli A. (2007). Noradrenaline in Parkinson’s disease: From disease progression to current therapeutics. Curr. Med. Chem..

[70] Espay A.J., LeWitt P.A., Kaufmann H. (2014). Norepinephrine deficiency in Parkinson’s disease: The case for noradrenergic enhancement. Mov. Disord..

[71] Feinstein D.L., Kalinin S., Braun D. (2016). Causes, consequences, and cures for neuroinflammation mediated via the locus coeruleus: Noradrenergic signaling system. J. Neurochem..

[72] Mani S., Sevanan M., Krishnamoorthy A., Sekar S. (2021). A systematic review of molecular approaches that link mitochondrial dysfunction and neuroinflammation in Parkinson’s disease. Neurol. Sci..

[73] Saravanan J. (2021). Role of microgliosis, oxidative stress and associated neuroinflammation in the pathogenesis of Parkinson’s disease: The therapeutic role of Nrf2 activators. Neurochem. Int..

[74] Spano M., Signorelli M., Vitaliani R., Giometto B. (2015). The possible involvement of mitochondrial dysfunctions in Lewy body dementia A systematic Review. Funct. Neurol..

[75] Surmeier D.J., Obeso J.A., Halliday G.M. (2017). Selective neuronal vulnerability in Parkinson disease. Nat. Rev. Neurosci..

[76] Burre J., Sharma M., Sudhof T.C. (2018). Cell Biology and Pathophysiology of alpha-Synuclein. Cold Spring Harb. Perspect. Med..

[77] Fisher A.B., Zhang Q., Laurent G.J., Shapiro S.D. (2006). NADPH and NADPH Oxidase. Encyclopedia of Respiratory Medicine.

[78] Zhang W., Wang T., Pei Z., Miller D.S., Wu X., Block M.L., Wilson B., Zhang W., Zhou Y., Hong J.S. (2005). Aggregated alpha-synuclein activates microglia: A process leading to disease progression in Parkinson’s disease. FASEB J..

[79] Bernheimer H., Birkmayer W., Hornykiewicz O., Jellinger K., Seitelberger F. (1973). Brain dopamine and the syndromes of Parkinson and Huntington. Clinical, morphological and neurochemical correlations. J. Neurol. Sci..

[80] Damier P., Hirsch E.C., Agid Y., Graybiel A.M. (1999). The substantia nigra of the human brain. II. Patterns of loss of dopamine-containing neurons in Parkinson’s disease. Brain.

[81] Zhang S., Wang R.F., Wang G. (2018). Impact of dopamine oxidation on dopaminergic neurodegeneration. ACS Chem..

[82] Hirsch E.C., Hunot S., Damier P., Faucheux B. (1998). Glial cells and inflammation in Parkinson’s disease: A role in neurodegeneration?. Ann. Neurol..

[83] Hirsch E., Graybiel A.M., Agid Y.A. (1988). Melanized dopaminergic neurons are differentially susceptible to degeneration in Parkinson’s disease. Nature.

[84] Pacelli C., Giguere N., Bourque M.J., Levesque M., Slack R.S., Trudeau L.E. (2015). Elevated Mitochondrial Bioenergetics and Axonal Arborization Size Are Key Contributors to the Vulnerability of Dopamine Neurons. Curr. Biol..

[85] Barja G. (2014). The Mitochondrial Free Radical Theory of Aging. Prog. Mol. Biol. Transl. Sci..

[86] Sun N., Youle R.J., Finkel T. (2016). The Mitochondrial Basis of Aging. Mol. Cell.

[87] Bratic A., Larsson N.G. (2013). The role of mitochondria in aging. J. Clin. Investig..

[88] Cassarino D.S., Bennett J.P. (1999). An evaluation of the role of mitochondria in neurodegenerative diseases: Mitochondrial mutations and oxidative pathology, protective nuclear responses, and cell death in neurodegeneration. Brain Res. Rev..

[89] Porter C., Hurren N.M., Cotter M.V., Bhattarai N., Reidy P.T., Dillon E.L., Durham W.J., Tuvdendorj D., Sheffield-Moore M., Volpi E. (2015). Mitochondrial respiratory capacity and coupling control decline with age in human skeletal muscle. Am. J. Physiol. Endocrinol. Metab..

[90] Zsurka G., Kunz W.S. (2013). Mitochondrial involvement in neurodegenerative diseases. IUBMB Life.

[91] Navarro A., Boveris A. (2010). Brain mitochondrial dysfunction in aging, neurodegeneration, and Parkinson’s disease. Front. Aging Neurosci..

[92] Rango M., Bresolin N. (2018). Brain Mitochondria, Aging, and Parkinson’s Disease. Genes.

[93] Fernie A.R., Carrari F., Sweetlove L.J. (2004). Respiratory metabolism: Glycolysis, the TCA cycle and mitochondrial electron transport. Curr. Opin. Plant Biol..

[94] Smeitink J., van den Heuvel L., DiMauro S. (2001). The genetics and pathology of oxidative phosphorylation. Nat. Rev. Genet..

[95] Westermann B. (2012). Bioenergetic role of mitochondrial fusion and fission. Biochim. Biophys. Acta (BBA) Bioenerg..

[96] Aldana B.I. (2019). Microglia-Specific Metabolic Changes in Neurodegeneration. J. Mol. Biol..

[97] Shin Y., Ryall J., Britto J., Lau C., Devenish R., Nagley P., Beart P. (2019). Inhibition of bioenergetics provides novel insights into recruitment of PINK 1-dependent neuronal mitophagy. J. Neurochem..

[98] Tait S.W., Green D.R. (2012). Mitochondria and cell signalling. J. Cell Sci..

[99] Zhou Z., Austin G.L., Young L.E.A., Johnson L.A., Sun R. (2018). Mitochondrial Metabolism in Major Neurological Diseases. Cells.

[100] Wai T., Langer T. (2016). Mitochondrial Dynamics and Metabolic Regulation. Trends Endocrinol. Metab..

[101] Dias V., Junn E., Mouradian M.M. (2013). The role of oxidative stress in Parkinson’s disease. J. Park. Dis..

[102] Salmina A.B., Kharitonova E.V., Gorina Y.V., Teplyashina E.A., Malinovskaya N.A., Khilazheva E.D., Mosyagina A.I., Morgun A.V., Shuvaev A.N., Salmin V.V. (2021). Blood-Brain Barrier and Neurovascular Unit In Vitro Models for Studying Mitochondria-Driven Molecular Mechanisms of Neurodegeneration. Int. J. Mol. Sci..

[103] Agnihotri A., Aruoma O.I. (2019). Alzheimer’s Disease and Parkinson’s Disease: A Nutritional Toxicology Perspective of the Impact of Oxidative Stress, Mitochondrial Dysfunction, Nutrigenomics and Environmental Chemicals. J. Am. Coll. Nutr..

[104] Kumar Sahel D., Kaira M., Raj K., Sharma S., Singh S. (2019). Mitochondrial dysfunctioning and neuroinflammation: Recent highlights on the possible mechanisms involved in Traumatic Brain Injury. Neurosci. Lett..

[105] Sorrentino V., Menzies K.J., Auwerx J. (2018). Repairing Mitochondrial Dysfunction in Disease. Annu. Rev. Pharm. Toxicol..

[106] Bose A., Beal M.F. (2016). Mitochondrial dysfunction in Parkinson’s disease. J. Neurochem..

[107] Duarte J.N. (2021). Neuroinflammatory Mechanisms of Mitochondrial Dysfunction and Neurodegeneration in Glaucoma. J. Ophthalmol..

[108] Song S., Meng R., Zhao X., Hua C., Kang K., Han Y., Ma X. (2019). Clinical significance of baseline neutrophil to lymphocyte ratio in patients with ischemic stroke or haemarragic stroke—An updated Meta-Analysis. Front. Neurol..

[109] Keane P.C., Kurzawa M., Blain P.G., Morris C.M. (2011). Mitochondrial dysfunction in Parkinson’s disease. Park. Dis..

[110] McGuire P.J. (2019). Mitochondrial Dysfunction and the Aging Immune System. Biology.

[111] Kapnick S.M., Pacheco S.E., McGuire P.J. (2018). The emerging role of immune dysfunction in mitochondrial diseases as a paradigm for understanding immunometabolism. Metabolism.

[112] Culmsee C., Michels S., Scheu S., Arolt V., Dannlowski U., Alferink J. (2018). Mitochondria, Microglia, and the Immune System-How Are They Linked in Affective Disorders?. Front. Psychiatry.

[113] Ploumi C., Daskalaki I., Tavernarakis N. (2017). Mitochondrial biogenesis and clearance: A balancing act. FEBS J..

[114] Lagouge M., Larsson N.G. (2013). The role of mitochondrial DNA mutations and free radicals in disease and ageing. J. Intern. Med..

[115] Turrens J.F. (2003). Mitochondrial formation of reactive oxygen species. J. Physiol..

[116] Ahmad M.H., Fatima M., Mondal A.C. (2019). Influence of microglia and astrocyte activation in the neuroinflammatory pathogenesis of Alzheimer’s disease: Rational insights for the therapeutic approaches. J. Clin. Neurosci..

[117] Grünewald A., Kumar K.R., Sue C.M. (2019). New insights into the complex role of mitochondria in Parkinson’s disease. Prog. Neurobiol..

[118] Takeshige K., Minakami S. (1979). NADH- and NADPH-dependent formation of superoxide anions by bovine heart submitochondrial particles and NADH-ubiquinone reductase preparation. Biochem. J..

[119] Van Horssen J., van Schaik P., Witte M. (2019). Inflammation and mitochondrial dysfunction: A vicious circle in neurodegenerative disorders?. Neurosci. Lett..

[120] Westermann B. (2010). Mitochondrial fusion and fission in cell life and death. Nat. Rev. Mol. Cell Biol..

[121] Chan D.C. (2012). Fusion and fission: Interlinked processes critical for mitochondrial health. Annu. Rev. Genet..

[122] Liu X.L., Wang Y.D., Yu X.M., Li D.W., Li G.R. (2018). Mitochondria-mediated damage to dopaminergic neurons in Parkinson’s disease (Review). Int. J. Mol. Med..

[123] Annesley S.J., Fisher P.R. (2019). Mitochondria in Health and Disease. Cells.

[124] Picca A., Calvani R., Coelho-Junior H.J., Landi F., Bernabei R., Marzetti E. (2020). Mitochondrial Dysfunction, Oxidative Stress, and Neuroinflammation: Intertwined Roads to Neurodegeneration. Antioxidants.

[125] Protasoni M., Zeviani M. (2021). Mitochondrial Structure and Bioenergetics in Normal and Disease Conditions. Int. J. Mol. Sci..

[126] Zhao X.Y., Lu M.H., Yuan D.J., Xu D.E., Yao P.P., Ji W.L., Chen H., Liu W.L., Yan C.X., Xia Y.Y. (2019). Mitochondrial Dysfunction in Neural Injury. Front. Neurosci..

[127] Park J., Choi H., Min J.S., Park S.J., Kim J.H., Park H.J., Kim B., Chae J.I., Yim M., Lee D.S. (2013). Mitochondrial dynamics modulate the expression of pro-inflammatory mediators in microglial cells. J. Neurochem..

[128] Garland E.F., Hartnell I.J., Boche D. (2022). Microglia and Astrocyte Function and Communication: What Do We Know in Humans?. Front. Neurosci..

[129] Harry G., McPherson C. (2014). Microglia: Neuroprotective and Neurodestructive Properties. Handbook of Neurotoxicity.

[130] Aires I.D., Ribeiro-Rodrigues T., Boia R., Ferreira-Rodrigues M., Girão H., Ambrósio A.F., Santiago A.R. (2021). Microglial extracellular vesicles as vehicles for neurodegeneration spreading. Biomolecules.

[131] Blaylock R.L. (2017). Parkinson’s disease: Microglial/macrophage-induced immunoexcitotoxicity as a central mechanism of neurodegeneration. Surg. Neurol. Int..

[132] Gonzalez H., Elgueta D., Montoya A., Pacheco R. (2014). Neuroimmune regulation of microglial activity involved in neuroinflammation and neurodegenerative diseases. J. Neuroimmunol..

[133] Amor S., Peferoen L.A., Vogel D.Y., Breur M., van der Valk P., Baker D., van Noort J.M. (2014). Inflammation in neurodegenerative diseases:an update. Immunology.

[134] Ghosh S., Castillo E., Frias E.S., Swanson R.A. (2018). Bioenergetic regulation of microglia. Glia.

[135] Bagheri H., Ghasemi F., Barreto G.E., Sathyapalan T., Jamialahmadi T., Sahebkar A. (2019). The effects of statins on microglial cells to protect against neurodegenerative disorders: A mechanistic review. Biofactors.

[136] Frank-Cannon T.C., Alto L.T., McAlpine F.E., Tansey M.G. (2009). Does neuroinflammation fan the flame in neurodegenerative diseases?. Mol. Neurodegener..

[137] Ren M., Guo Y., Wei X., Yan S., Qin Y., Zhang X., Jiang F., Lou H. (2018). TREM2 overexpression attenuates neuroinflammation and protects dopaminergic neurons in experimental models of Parkinson’s disease. Exp. Neurol..

[138] Block M.L., Zecca L., Hong J.S. (2007). Microglia-mediated neurotoxicity: Uncovering the molecular mechanisms. Nat. Rev. Neurosci..

[139] Wu A.G., Zhou X.G., Qiao G., Yu L., Tang Y., Yan L., Qiu W.Q., Pan R., Yu C.L., Law B.Y. (2021). Targeting microglial autophagic degradation in NLRP3 inflammasome-mediated neurodegenerative diseases. Ageing Res. Rev..

[140] Haque M.E., Akther M., Jakaria M., Kim I.S., Azam S., Choi D.K. (2019). Targeting the microglial NLRP3 inflammasome and its role in Parkinson’s disease. Mov. Disord..

[141] Pisanu A., Lecca D., Mulas G., Wardas J., Simbula G., Spiga S., Carta A.R. (2014). Dynamic changes in pro- and anti-inflammatory cytokines in microglia after PPAR-gamma agonist neuroprotective treatment in the MPTPp mouse model of progressive Parkinson’s disease. Neurobiol. Dis..

[142] Ouchi Y., Yoshikawa E., Sekine Y., Futatsubashi M., Kanno T., Ogusu T., Torizuka T. (2005). Microglial activation and dopamine terminal loss in early Parkinson’s disease. Ann. Neurol..

[143] Qin X.Y., Zhang S.P., Cao C., Loh Y.P., Cheng Y. (2016). Aberrations in Peripheral Inflammatory Cytokine Levels in Parkinson Disease: A Systematic Review and Meta-analysis. JAMA Neurol..

[144] Wang Q., Liu Y., Zhou J. (2015). Neuroinflammation in Parkinson’s disease and its potential as therapeutic target. Transl. Neurodegener..

[145] Süβ P., Lana A.J., Schlachetzki J.C.M. (2021). Chronic peripheral inflammation: A possible contributor to neurodegenerative diseases. Neural Regen. Res..

[146] Ponce J., Ulu A., Hanson C., Cameron-Smith E., Bertoni J., Wuebker J., Fisher A., Siu K.C., Marmelat V., Adamec J. (2022). Role of Specialized Pro-resolving Mediators in Reducing Neuroinflammation in Neurodegenerative Disorders. Front. Aging Neurosci..

[147] Serhan C.N. (2014). Pro-resolving lipid mediators are leads for resolution physiology. Nature.

[148] Xu J., Gao X., Yang C., Chen L., Chen Z. (2017). Resolvin D1 Attenuates Mpp+-Induced Parkinson Disease via Inhibiting Inflammation in PC12 Cells. Med. Sci. Monit..

[149] Polymenidou M., Cleveland D.W. (2012). Prion-like spread of protein aggregates in neurodegeneration. J. Exp. Med..

[150] Freeman D., Cedillos R., Choyke S., Lukic Z., McGuire K., Marvin S., Burrage A.M., Sudholt S., Rana A., O’Connor C. (2013). Alpha-synuclein induces lysosomal rupture and cathepsin dependent reactive oxygen species following endocytosis. PLoS ONE.

[151] Shahmoradian S.H., Lewis A.J., Genoud C., Hench J., Moors T.E., Navarro P.P., Castano-Diez D., Schweighauser G., Graff-Meyer A., Goldie K.N. (2019). Lewy pathology in Parkinson’s disease consists of crowded organelles and lipid membranes. Nat. Neurosci..

[152] Guardia-Laguarta C., Area-Gomez E., Rüb C., Liu Y., Magrané J., Becker D., Voos W., Schon E.A., Przedborski S. (2014). α-Synuclein is localized to mitochondria-associated ER membranes. J. Neurosci..

[153] Rehman M.U., Sehar N., Dar N.J., Khan A., Arafah A., Rashid S., Rashid S.M., Ganaie M.A. (2023). Mitochondrial dysfunctions, oxidative stress and neuroinflammation as therapeutic targets for neurodegenerative diseases: An update on current advances and impediments. Neurosci. Biobehav. Rev..

[154] Carta S., Penco F., Lavieri R., Martini A., Dinarello C.A., Gattorno M., Rubartelli A. (2015). Cell stress increases ATP release in NLRP3 inflammasome-mediated autoinflammatory diseases, resulting in cytokine imbalance. Proc. Natl. Acad. Sci. USA.

[155] Wu Y., Yao Q., Jiang G.X., Wang G., Cheng Q. (2020). Identification of distinct blood-based biomarkers in early stage of Parkinson’s disease. Neurol. Sci..

[156] George S., Rey N.L., Tyson T., Esquibel C., Meyerdirk L., Schulz E., Pierce S., Burmeister A.R., Madaj Z., Steiner J.A. (2019). Microglia affect alpha-synuclein cell-to-cell transfer in a mouse model of Parkinson’s disease. Mol. Neurodegener..

[157] Nonaka T., Watanabe S.T., Iwatsubo T., Hasegawa M. (2010). Seeded aggregation and toxicity of {alpha}-synuclein and tau: Cellular models of neurodegenerative diseases. J. Biol. Chem..

[158] Mosser D.M., Edwards J.P. (2008). Exploring the full spectrum of macrophage activation. Nat. Rev. Immunol..

[159] Wolf S.A., Boddeke H.W., Kettenmann H. (2017). Microglia in Physiology and Disease. Annu. Rev. Physiol..

[160] Tan Y.L., Yuan Y., Tian L. (2020). Microglial regional heterogeneity and its role in the brain. Mol. Psychiatry.

[161] Hammond T.R., Dufort C., Dissing-Olesen L., Giera S., Young A., Wysoker A., Walker A.J., Gergits F., Segel M., Nemesh J. (2019). Single-Cell RNA Sequencing of Microglia throughout the Mouse Lifespan and in the Injured Brain Reveals Complex Cell-State Changes. Immunity.

[162] Hanisch U.K. (2013). Functional diversity of microglia–how heterogeneous are they to begin with?. Front. Cell. Neurosci..

[163] Hanisch U.K., Kettenmann H. (2007). Microglia: Active sensor and versatile effector cells in the normal and pathologic brain. Nat. Neurosci..

[164] Simpson D.S.A., Oliver P.L. (2020). ROS Generation in Microglia: Understanding Oxidative Stress and Inflammation in Neurodegenerative Disease. Antioxid. Redox Signal..

[165] Chausse B., Lewen A., Poschet G., Kann O. (2020). Selective inhibition of mitochondrial respiratory complexes controls the transition of microglia into a neurotoxic phenotype in situ. Brain Behav. Immun..

[166] Gertig U., Hanisch U.K. (2014). Microglial diversity by responses and responders. Front. Cell. Neurosci..

[167] Cauwels A., Rogge E., Vandendriessche B., Shiva S., Brouckaert P. (2014). Extracellular ATP drives systemic inflammation, tissue damage and mortality. Cell Death Dis..

[168] Tansey M.G., Goldberg M.S. (2010). Neuroinflammation in Parkinson’s disease: Its role in neuronal death and implications for therapeutic intervention. Neurobiol. Dis..

[169] Rodriguez-Iturbe B., Pons H., Johnson R.J. (2017). Role of the Immune System in Hypertension. Physiol. Rev..

[170] Di Stadio A., Angelini C. (2019). Microglia polarization by mitochondrial metabolism modulation: A therapeutic opportunity in neurodegenerative diseases. Mitochondrion.

[171] Smith J.A., Das A., Ray S.K., Banik N.L. (2012). Role of pro-inflammatory cytokines released from microglia in neurodegenerative diseases. Brain Res. Bull..

[172] Subhramanyam C.S., Wang C., Hu Q., Dheen S.T. (2019). Microglia-mediated neuroinflammation in neurodegenerative diseases. Semin. Cell Dev. Biol..

[173] Lawson L.J., Perry V.H., Dri P., Gordon S. (1990). Heterogeneity in the distribution and morphology of microglia in the normal adult mouse brain. Neuroscience.

[174] Rojo A.I., Innamorato N.G., Martin-Moreno A.M., De Ceballos M.L., Yamamoto M., Cuadrado A. (2010). Nrf2 regulates microglial dynamics and neuroinflammation in experimental Parkinson’s disease. Glia.

[175] Ward R.J., Dexter D.T., Crichton R.R. (2015). Ageing, neuroinflammation and neurodegeneration. Front. Biosci..

[176] Heneka M.T., McManus R.M., Latz E. (2018). Inflammasome signalling in brain function and neurodegenerative disease. Nat. Rev. Neurosci..

[177] Heneka M.T., Kummer M.P., Latz E. (2014). Innate immune activation in neurodegenerative disease. Nat. Rev. Immunol..

[178] Kempuraj D., Thangavel R., Selvakumar G.P., Zaheer S., Ahmed M.E., Raikwar S.P., Zahoor H., Saeed D., Natteru P.A., Iyer S. (2017). Brain and Peripheral Atypical Inflammatory Mediators Potentiate Neuroinflammation and Neurodegeneration. Front. Cell Neurosci..

[179] Litteljohn D., Mangano E., Clarke M., Bobyn J., Moloney K., Hayley S. (2011). Inflammatory mechanisms of neurodegeneration in toxin-based models of Parkinson’s disease. Park. Dis..

[180] Fernandez-Botran R., Ahmed Z., Crespo F.A., Gatenbee C., Gonzalez J., Dickson D.W., Litvan I. (2011). Cytokine expression and microglial activation in progressive supranuclear palsy. Park. Relat. Disord..

[181] Pal R., Tiwari P.C., Nath R., Pant K.K. (2016). Role of neuroinflammation and latent transcription factors in pathogenesis of Parkinson’s disease. Neurol. Res..

[182] Kaur K., Gill J.S., Bansal P.K., Deshmukh R. (2017). Neuroinflammation—A major cause for striatal dopaminergic degeneration in Parkinson’s disease. J. Neurol. Sci..

[183] Perry V.H., Teeling J. (2013). Microglia and macrophages of the central nervous system: The contribution of microglia priming and systemic inflammation to chronic neurodegeneration. Semin. Immunopathol..

[184] Davis A.A., Inman C.E., Wargel Z.M., Dube U., Freeberg B.M., Galluppi A., Haines J.N., Dhavale D.D., Miller R., Choudhury F.A. (2020). APOE genotype regulates pathology and disease progression in synucleinopathy. Sci. Transl. Med..

[185] Smith J.D. (2000). Apolipoprotein E4: An allele associated with many diseases. Ann. Med..

[186] De Lau L.M., Koudstaal P.J., Hofman A., Breteler M.M. (2006). Serum cholesterol levels and the risk of Parkinson’s disease. Am. J. Epidemiol..

[187] Williams-Gray C., Goris A., Saiki M., Foltynie T., Compston D.A., Sawcer S.J., Barker R.A. (2009). Apolipoprotein E genotype as a risk factor for susceptibility to and dementia in Parkinson’s disease. J. Neurol..

[188] Gao J., Huang X., Park Y., Liu R., Hollenbeck A., Schatzkin A., Mailman R.B., Chen H. (2011). Apolipoprotein E genotypes and the risk of Parkinson disease. Neurobiol. Aging.

[189] Pitas R.E., Boyles J.K., Lee S.H., Foss D., Mahley R.W. (1987). Astrocytes synthesize apolipoprotein E and metabolize apolipoprotein E-containing lipoproteins. Biochim. Biophys. Acta (BBA) Lipids Lipid Metab..

[190] Wagner J., Neher J.J. (2021). Killing ageing neurons, one cell at a time. Nat. Neurosci..

[191] Davis R.L., Wong S.L., Carling P.J., Payne T., Sue C.M., Bandmann O. (2020). Serum FGF-21, GDF-15, and blood mtDNA copy number are not biomarkers of Parkinson disease. Neurol. Clin. Pract..

[192] Zalocusky K.A., Najm R., Taubes A.L., Hao Y., Yoon S.Y., Koutsodendris N., Nelson M.R., Rao A., Bennett D.A., Bant J. (2021). Neuronal ApoE upregulates MHC-I expression to drive selective neurodegeneration in Alzheimer’s disease. Nat. Neurosci..

[193] Simonovitch S., Schmukler E., Masliah E., Pinkas-Kramarski R., Michaelson D.M. (2019). The Effects of APOE4 on Mitochondrial Dynamics and Proteins in vivo. J. Alzheimers Dis..

[194] Cebrian C., Zucca F.A., Mauri P., Steinbeck J.A., Studer L., Scherzer C.R., Kanter E., Budhu S., Mandelbaum J., Vonsattel J.P. (2014). MHC-I expression renders catecholaminergic neurons susceptible to T-cell-mediated degeneration. Nat. Commun..

[195] Neumann H., Cavalie A., Jenne D.E., Wekerle H. (1995). Induction of MHC class I genes in neurons. Science.

[196] Schmukler E., Solomon S., Simonovitch S., Goldshmit Y., Wolfson E., Michaelson D.M., Pinkas-Kramarski R. (2020). Altered mitochondrial dynamics and function in APOE4-expressing astrocytes. Cell Death Disord..

[197] Dose J., Huebbe P., Nebel A., Rimbach G. (2016). APOE genotype and stress response-a mini review. Lipids Health Dis..

[198] Nakamura T., Watanabe A., Fujino T., Hosono T., Michikawa M. (2009). Apolipoprotein E4 (1-272) fragment is associated with mitochondrial proteins and affects mitochondrial function in neuronal cells. Mol. Neurodegener..

[199] Chen H.K., Ji Z.S., Dodson S.E., Miranda R.D., Rosenblum C.I., Reynolds I.J., Freedman S.B., Weisgraber K.H., Huang Y.D., Mahley R.W. (2011). Apolipoprotein E4 Domain Interaction Mediates Detrimental Effects on Mitochondria and Is a Potential Therapeutic Target for Alzheimer Disease. J. Biol. Chem..

[200] Yin J., Reiman E.M., Beach T.G., Serrano G.E., Sabbagh M.N., Nielsen M., Caselli R.J., Shi J. (2020). Effect of ApoE isoforms on mitochondria in Alzheimer disease. Neurology.

[201] Azam S., Haque M.E., Kim I.S., Choi D.K. (2021). Microglial Turnover in Ageing-Related Neurodegeneration: Therapeutic Avenue to Intervene in Disease Progression. Cells.

[202] Oberg M., Fabrik I., Fabrikova D., Zehetner N., Hartlova A. (2021). The role of innate immunity and inflammation in Parkinson s disease. Scand. J. Immunol..

[203] Currais A., Fischer W., Maher P., Schubert D. (2017). Intraneuronal protein aggregation as a trigger for inflammation and neurodegeneration in the aging brain. FASEB J..

[204] Currais A. (2015). Ageing and inflammation—A central role for mitochondria in brain health and disease. Ageing Res. Rev..

[205] Schain M., Kreisl W.C. (2017). Neuroinflammation in Neurodegenerative Disorders-a Review. Curr. Neurol. Neurosci. Rep..

[206] Lenaz G., D’Aurelio M., Merlo Pich M., Genova M.L., Ventura B., Bovina C., Formiggini G., Parenti Castelli G. (2000). Mitochondrial bioenergetics in aging. Biochim. Biophys. Acta (BBA) Bioenerg..

[207] Chandra G., Shenoi R., Anand R., Rajamma U., Mohanakumar K. (2019). Reinforcing mitochondrial functions in aging brain: An insight into Parkinson’s disease therapeutics. J. Chem. Neuroanat..

[208] Pence B.D., Yarbro J.R. (2018). Aging impairs mitochondrial respiratory capacity in classical monocytes. Exp. Gerontol..

[209] Witte M.E., Geurts J.J., de Vries H.E., van der Valk P., van Horssen J. (2010). Mitochondrial dysfunction: A potential link between neuroinflammation and neurodegeneration?. Mitochondrion.

[210] Bajwa E., Pointer C.B., Klegeris A. (2019). The Role of Mitochondrial Damage-Associated Molecular Patterns in Chronic Neuroinflammation. Mediat. Inflamm..

[211] Trudler D., Nash Y., Frenkel D. (2015). New insights on Parkinson’s disease genes: The link between mitochondria impairment and neuroinflammation. J. Neural. Transm..

[212] Riddell N., Crewther S.G. (2017). Novel evidence for complement system activation in chick myopia and hyperopia models: A meta-analysis of transcriptome datasets. Science.

[213] Riddell N., Faou P., Murphy M., Giummarra L., Downs R.A., Rajapaksha H., Crewther S.G. (2017). The retina/RPE proteome in chick myopia and hyperopia models: Commonalities with inherited and age-related ocular pathologies. Mol. Vis..

[214] Sun F., Deng Y., Han X., Liu Q., Zhang P., Manzoor R., Ma H. (2019). A secret that underlies Parkinson’s disease: The damaging cycle. Neurochem. Int..

[215] Franceschi C., Capri M., Monti D., Giunta S., Olivieri F., Sevini F., Panourgia M.P., Invidia L., Celani L., Scurti M. (2007). Inflammaging and anti-inflammaging: A systemic perspective on aging and longevity emerged from studies in humans. Mech. Ageing Dev..

[216] Gyengesi E., Rangel A., Ullah F., Liang H., Niedermayer G., Asgarov R., Venigalla M., Gunawardena D., Karl T., Munch G. (2019). Chronic Microglial Activation in the GFAP-IL6 Mouse Contributes to Age-Dependent Cerebellar Volume Loss and Impairment in Motor Function. Front. Neurosci..

[217] Meszaros A., Molnar K., Nogradi B., Hernadi Z., Nyul-Toth A., Wilhelm I., Krizbai I.A. (2020). Neurovascular Inflammaging in Health and Disease. Cells.

[218] Calabrese V., Santoro A., Monti D., Crupi R., Di Paola R., Latteri S., Cuzzocrea S., Zappia M., Giordano J., Calabrese E.J. (2018). Aging and Parkinson’s Disease: Inflammaging, neuroinflammation and biological remodeling as key factors in pathogenesis. Free Radic. Biol. Med..

[219] Tronel C., Largeau B., Santiago Ribeiro M.J., Guilloteau D., Dupont A.C., Arlicot N. (2017). Molecular Targets for PET Imaging of Activated Microglia: The Current Situation and Future Expectations. Int. J. Mol. Sci..

[220] Franceschi C., Campisi J. (2014). Chronic inflammation (inflammaging) and its potential contribution to age-associated diseases. J. Gerontol. Ser. A Biomed. Sci. Med. Sci..

[221] Plaza-Zabala A., Sierra-Torre V., Sierra A. (2017). Autophagy and Microglia: Novel Partners in Neurodegeneration and Aging. Int. J. Mol. Sci..

[222] Harry G.J. (2013). Microglia during development and aging. Pharmacol. Ther..

[223] Santoro A., Spinelli C.C., Martucciello S., Nori S.L., Capunzo M., Puca A.A., Ciaglia E. (2018). Innate immunity and cellular senescence: The good and the bad in the developmental and aged brain. J. Leukoc. Biol..

[224] Fulop T., Larbi A., Dupuis G., Le Page A., Frost E.H., Cohen A.A., Witkowski J.M., Franceschi C. (2018). Immunosenescence and inflammaging as two sides of the same coin: Friends or foes?. Front. Immunol..

[225] Dupré-Crochet S., Erard M., Nüβe O. (2013). ROS production in phagocytes: Why, when, and where?. J. Leukoc. Biol..

[226] Bournat J.C., Brown C.W. (2010). Mitochondrial dysfunction in obesity. Curr. Opin. Endocrinol. Diabetes Obes..

[227] Krief S., Lonnqvist F., Raimbault S., Baude B., Van Spronsen A., Arner P., Strosberg A.D., Ricquier D., Emorine L.J. (1993). Tissue distribution of beta 3-adrenergic receptor mRNA in man. J. Clin. Investig..

[228] Santiago J.A., Bottero V., Potashkin J.A. (2017). Biological and Clinical Implications of Comorbidities in Parkinson’s Disease. Front. Aging Neurosci..

[229] Santiago J.A., Potashkin J.A. (2014). System-based approaches to decode the molecular links in Parkinson’s disease and diabetes. Neurobiol. Dis..

[230] Fuger P., Hefendehl J.K., Veeraraghavalu K., Wendeln A.C., Schlosser C., Obermuller U., Wegenast-Braun B.M., Neher J.J., Martus P., Kohsaka S. (2017). Microglia turnover with aging and in an Alzheimer’s model via long-term in vivo single-cell imaging. Nat. Neurosci..

[231] Presta I., Vismara M., Novellino F., Donato A., Zaffino P., Scali E., Pirrone K.C., Spadea M.F., Malara N., Donato G. (2018). Innate Immunity Cells and the Neurovascular Unit. Int. J. Mol. Sci..

[232] Watson E., Davis R., Sue C.M. (2020). New diagnostic pathways for mitochondrial disease. J. Transl. Genet. Genom..

[233] Wallace D.C. (1999). Mitochondrial diseases in man and mouse. Science.

[234] Wallace D.C. (2005). A mitochondrial paradigm of metabolic and degenerative diseases, aging, and cancer: A dawn for evolutionary medicine. Annu. Rev. Genet..

[235] Gorman G.S., Chinnery P.F., DiMauro S., Hirano M., Koga Y., McFarland R., Suomalainen A., Thorburn D.R., Zeviani M., Turnbull D.M. (2016). Mitochondrial diseases. Nat. Rev. Dis. Prim..

[236] Schapira A.H. (2006). Mitochondrial disease. Lancet.

[237] Schapira A.H. (2007). Mitochondrial dysfunction in Parkinson’s disease. Cell Death Differ..

[238] Schapira A.H., Gegg M. (2011). Mitochondrial contribution to Parkinson’s disease pathogenesis. Park. Dis..

[239] Edmonds J.L. (2002). The otolaryngological manifestations of mitochondrial disease and the risk of neurodegeneration with infection. (Archives of Otolaryngology—Head & Neck Surgery). JAMA J. Am. Med. Assoc..

[240] Brand M.D., Nicholls D.G. (2011). Assessing mitochondrial dysfunction in cells. Biochem. J..

[241] Deeb W., Nozile-Firth K., Okun M.S. (2019). Parkinson’s disease: Diagnosis and appreciation of comorbidities. Handb. Clin. Neurol..

[242] Yu J.W., Walter B.L. (2022). Addressing critical care gaps in inpatient Parkinson’s care–Minimizing the impact of comorbidities and developing new care delivery models. Park. Relat. Disord..

[243] Joshi A.U., Minhas P.S., Liddelow S.A., Haileselassie B., Andreasson K.I., Dorn G.W., Mochly-Rosen D. (2019). Fragmented mitochondria released from microglia trigger A1 astrocytic response and propagate inflammatory neurodegeneration. Nat. Neurosci..

[244] Rabaneda-Lombarte N., Xicoy-Espaulella E., Serratosa J., Saura J., Sola C. (2018). Parkinsonian Neurotoxins Impair the Pro-inflammatory Response of Glial Cells. Front. Mol. Neurosci..

[245] Moya G.E., Rivera P.D., Dittenhafer-Reed K.E. (2021). Evidence for the Role of Mitochondrial DNA Release in the Inflammatory Response in Neurological Disorders. Int. J. Mol. Sci..

[246] Harper M.E., Monemdjou S., Ramsey J.J., Weindruch R. (1998). Age-related increase in mitochondrial proton leak and decrease in ATP turnover reactions in mouse hepatocytes. Am. J. Physiol.-Endocrinol. Metab..

[247] Akbar M., Essa M.M., Daradkeh G., Abdelmegeed M.A., Choi Y., Mahmood L., Song B.J. (2016). Mitochondrial dysfunction and cell death in neurodegenerative diseases through nitroxidative stress. Brain Res..

[248] Singh A., Kukreti R., Saso L., Kukreti S. (2019). Oxidative Stress: A Key Modulator in Neurodegenerative Diseases. Molecules.

[249] Islam M.T. (2017). Oxidative stress and mitochondrial dysfunction-linked neurodegenerative disorders. Neurol. Res..

[250] Sharma A., Liaw K., Sharma R., Zhang Z., Kannan S., Kannan R.M. (2018). Targeting Mitochondrial Dysfunction and Oxidative Stress in Activated Microglia Using Dendrimer-Based Therapeutics. Theranostics.

[251] Luna-Sánchez M., Bianchi P., Quintana A. (2021). Mitochondria-Induced Immune Response as a Trigger for Neurodegeneration: A Pathogen from Within. Int. J. Mol. Sci..

[252] Ferger A.I., Campanelli L., Reimer V., Muth K.N., Merdian I., Ludolph A.C., Witting A. (2010). Effects of mitochondrial dysfunction on the immunological properties of microglia. J. Neuroinflamm..

[253] Fang C., Wei X., Wei Y. (2016). Mitochondrial DNA in the regulation of innate immune responses. Protein Cell.

[254] Reichenbach J., Schubert R., Horvath R., Petersen J., Futterer N., Malle E., Stumpf A., Gebhardt B.R., Koehl U., Schraven B. (2006). Fatal neonatal-onset mitochondrial respiratory chain disease with T cell immunodeficiency. Pediatr. Res..

[255] Annesley S.J., Lay S.T., De Piazza S.W., Sanislav O., Hammersley E., Allan C.Y., Francione L.M., Bui M.Q., Chen Z.P., Ngoei K.R. (2016). Immortalized Parkinson’s disease lymphocytes have enhanced mitochondrial respiratory activity. Dis. Model. Mech..

[256] Requejo-Aguilar R., Bolanos J.P. (2016). Mitochondrial control of cell bioenergetics in Parkinson’s disease. Free Radic. Biol. Med..

[257] Scorziello A., Borzacchiello D., Sisalli M.J., Di Martino R., Morelli M., Feliciello A. (2020). Mitochondrial Homeostasis and Signaling in Parkinson’s Disease. Front. Aging Neurosci..

[258] Narendra D.P., Jin S., Tanaka A., Suen D., Gautier C., Shen J., Cookson M.R., Youle R.J. (2010). PINK1 is selectively stabilized on impaired mitochondria to activate Parkin. PLoS Biol..

[259] Elfawy H.A., Das B. (2019). Crosstalk between mitochondrial dysfunction, oxidative stress, and age related neurodegenerative disease: Etiologies and therapeutic strategies. Life Sci..

[260] Cheng X.T., Sheng Z.H. (2021). Developmental regulation of microtubule-based trafficking and anchoring of axonal mitochondria in health and diseases. Dev. Neurobiol..

[261] Ray B., Bhat A., Mahalakshmi A.M., Tuladhar S., Bishir M., Mohan S.K., Veeraraghavan V.P., Chandra R., Essa M.M., Chidambaram S.B. (2021). Mitochondrial and Organellar Crosstalk in Parkinson’s Disease. Am. Soc. Neurochem..

[262] Trushina E., McMurray C.T. (2007). Oxidative stress and mitochondrial dysfunction in neurodegenerative diseases. Neuroscience.

[263] Mishra A.K., Dixit A. (2022). Dopaminergic Axons: Key Recitalists in Parkinson’s Disease. Neurochem Res..

[264] Chen C., Turnbull D.M., Reeve A.K. (2019). Mitochondrial Dysfunction in Parkinson’s Disease-Cause or Consequence?. Biology.

[265] Landau S.M., Harvey D., Madison C.M., Reiman E.M., Foster N.L., Aisen P.S., Petersen R.C., Shaw L.M., Trojanowski J.Q., Jack C.R. (2010). Comparing predictors of conversion and decline in mild cognitive impairment. Neurology.

[266] Segura-Aguilar J., Paris I., Munoz P., Ferrari E., Zecca L., Zucca F.A. (2014). Protective and toxic roles of dopamine in Parkinson’s disease. J. Neurochem..

[267] Munoz-Manchado A.B., Villadiego J., Romo-Madero S., Suarez-Luna N., Bermejo-Navas A., Rodriguez-Gomez J.A., Garrido-Gil P., Labandeira-Garcia J.L., Echevarria M., Lopez-Barneo J. (2016). Chronic and progressive Parkinson’s disease MPTP model in adult and aged mice. J. Neurochem..

[268] van Ham T.J., Thijssen K.L., Breitling R., Hofstra R.M., Plasterk R.H., Nollen E.A.C. (2008). elegans model identifies genetic modifiers of alpha-synuclein inclusion formation during aging. PLoS Genet..

[269] Martinez T.N., Greenamyre J.T. (2012). Toxin models of mitochondrial dysfunction in Parkinson’s disease. Antioxid Redox Signal..

[270] Ransohoff R.M. (2016). How neuroinflammation contributes to neurodegeneration. Science.

[271] Langston J.W., Forno L.S., Tetrud J., Reeves A.G., Kaplan J.A., Karluk D. (1999). Evidence of active nerve cell degeneration in the substantia nigra of humans years after 1-methyl-4-phenyl-1,2,3,6-tetrahydropyridine exposure. Ann. Neurol..

[272] Kurihara K., Nakagawa R., Ishido M., Yoshinaga Y., Watanabe J., Hayashi Y., Mishima T., Fujioka S., Tsuboi Y. (2020). Impact of motor and nonmotor symptoms in Parkinson disease for the quality of life: The Japanese Quality-of-Life Survey of Parkinson Disease (JAQPAD) study. J. Neurol. Sci..

[273] Zhang L., Yang T., Chen Y., Zheng D., Sun D., Tu Q., Huang J., Zhang J., Li Z. (2022). Cognitive Deficit and Aberrant Intrinsic Brain Functional Network in Early-Stage Drug-Naive Parkinson’s Disease. Front. Neurosci..

[274] Baik S.H., Kang S., Lee W., Choi H., Chung S., Kim J.I., Mook-Jung I. (2019). A Breakdown in Metabolic Reprogramming Causes Microglia Dysfunction in Alzheimer’s Disease. Cell Metab..

[275] Holland R., McIntosh A.L., Finucane O.M., Mela V., Rubio-Araiz A., Timmons G., McCarthy S.A., Gun’ko Y.K., Lynch M.A. (2018). Inflammatory microglia are glycolytic and iron retentive and typify the microglia in APP/PS1 mice. Brain Behav. Immun..

[276] Ye J., Jiang Z., Chen X., Liu M., Li J., Liu N. (2016). Electron transport chain inhibitors induce microglia activation through enhancing mitochondrial reactive oxygen species production. Exp. Cell Res..

[277] Hunot S., Brugg B., Ricard D., Michel P.P., Muriel M.P., Ruberg M., Faucheux B.A., Agid Y., Hirsch E.C. (1997). Nuclear translocation of NF-kappaB is increased in dopaminergic neurons of patients with Parkinson disease. Proc. Natl. Acad. Sci. USA.

[278] Rasmussen M.K., Mestre H., Nedergaard M. (2018). The glymphatic pathway in neurological disorders. Lancet Neurol..

